# Treatment of the Third Stage of Alveolar Abscess

**Published:** 1891-02

**Authors:** E. C. Moore

**Affiliations:** Detroit, Mich.


ARTICLE II.

### TREATMENT OF THE THIRD STAGE OF AL-VEOLAR ABSCESS.

BY E. C. MOORE, D. D. S., DETROIT, MICH.

**[Read before the Michigan State Dental Society, 1890.]**

I am of the opinion that in almost every case of alveolar abscess the happiest results may be attained without the application of medicine, unless we may construe water as coming under that head. Although the writer generally uses some antiseptic or disinfectant, or, in the language of Dr. George Watt, "a stink disguiser," or germ destroyer,—this not so much as an element in the curative process, as for the comfort and pacification of his olfactories,—the treatment consists chiefly in removing the cause or source of this outflowing fountain, the putrescing nerve or pulp, just exactly as one would remove from the alley a dead and decomposing cat or dog if in too close proximity to his residence. It is of little consequence how this is accomplished, so that it is effectually accomplished—the source removed, eradicated; just a little fresh dirt thrown over the defunct animal may change the character of the air wafted through the family residence for a time, but as the elements of combustion, sun and air or heat and air, again reach the dead flesh, the product of decomposition is made manifest. Just so in the treatment of the nerve canal, the dead cat must not only be thoroughly removed, but a barrier placed against its possible return, and, so to speak, the alley closed up. This dead matter, animal or vegetable, must be removed, and in its stead a filling of almost any indestructible substance must thoroughly seal the apex of the root at least. Now, in the removal of this matter, and in the process of preparation for filling, there is one infallible remedy or preventive which I am going to disclose to you as a great secret, (and if it should be the only recommendation in this paper heeded, the writer is content,) and which, if you will use, can not fail to prevent trouble. We often hear practitioners speak of sure cures, or specifics for certain troubles, which we are apt to take with at least a grain of allowance; but I tell you I have a sure cure or preventive, and I can demonstrate to a certainty its infallibility, and that is a preventive of going through the side of the root in the process of cleansing or preparing for filling. Never, never, never use a drill in this connection; it is useless, it is unnecessary, it is bad, it is senseless, it is unpardonable, it is criminal, and should be a State prison offense, so inexcusable is it. If this precaution or preventive is acted upon, it is simply impossible to puncture the periosteum, unless there is an opening already there, for which you can in no way be responsible, and here is the strong point in favor of this advice. If you do not use the drill, you cannot be held or made responsible for any openings through the root. You are satisfied in your own conscience that you have not at any point gone through the side or bottom. With the excellent quality of broaches and canal cleaners, particularly the Donaldson, there is no excuse, and no guilty man should escape.

As I have already intimated in this short paper, it is of little consequence what the canal is filled with so long as the substance is indestructible in the mouth; but both the cleansing and the filling must be thorough—be sure of this. And to do all this it is unnecessary to wait a minute for farther treatment than has already been described in this paper. The permanent filling, so far as the canal is concerned, can be put in at once, and there is the end of it, leaving the rest to the tender mercies of dame nature, who only mixes a little time with her remedies.

For a filling material the writer prefers tin or oxychloride of zinc, the former being used in the straight, or single canaled teeth, and is prepared from a light number of foil by shaving off with a sharp pair of foil shears into hair-like strips. One end of one of these hair-like strips is placed at the orifice of the canal to be filled, and with a fine, smooth broach reduced to this fineness and smoothness by a fine oil stone, and with the same squared across the end, the foil is caught by this squared-end broach and carried to the extreme end of the canal, and by a slight backward and forward motion the rest of the strip is tucked in and made quite compact. This operation is repeated to the satisfaction of the operator, and in receding from the apex of the canal, and as it grows larger, larger instruments are used. It is not the intention of the writer to enter too much into minutiæ. All this is worked out by the operator himself.

Oxychloride of zinc is used in a class or kind of root filling where the canal is very small or difficult of access, or the canal can only be entered by using a curved instrument. A very fine broach, prepared as above described, only smaller, is used to work this cream-like mixture of oxychloride of zinc into the finest canals. A small amount of the mixture is placed at the orifice and the line broach punctures it and passes on to the extreme end of the canal, and then a slight motion of the instrument will cause the thin mixture to disappear into the canal, removing the in strument occasionally to expel the air from the canal, to allow the oxychloride to take its place. This is preferable, in the writer's estimation, to a solution of gutta-percha and chloroform; it is more liquid-like and does not stiffen like the gutta-percha, owing to the rapid evaporation of the chloroform.

After this short description, passing around some of these small instruments will give you a better idea how this kind of root filling is done, and how the little instruments are prepared.—*Ohio Journal of Dental Science.*

